# Male obesity effects on sperm and next-generation cord blood DNA methylation

**DOI:** 10.1371/journal.pone.0218615

**Published:** 2019-06-27

**Authors:** Ramya Potabattula, Marcus Dittrich, Martin Schorsch, Thomas Hahn, Thomas Haaf, Nady El Hajj

**Affiliations:** 1 Institute of Human Genetics, Julius Maximilians University, Würzburg, Germany; 2 Fertility Center; Wiesbaden, Germany; 3 College of Health and Life Sciences, Hamad Bin Khalifa University, Education City, Doha, Qatar; University of Oslo, NORWAY

## Abstract

The prevalence of metabolic disorders, in particular obesity has dramatically increased worldwide. Genetic variants explain only a minor part of the obesity epidemic induced by physical inactivity and over-nutrition. Epidemiological studies in humans and animal models indicate that epigenetic changes associated with adverse parental and/or intrauterine factors may contribute to the missing heritability of metabolic disorders. Possible adverse paternal effects are likely transmitted by sperm to the next-generation. To investigate this hypothesis, we have systematically analyzed the effects of male body mass index (BMI) on sperm epigenome and its association with next-generation fetal cord blood (FCB) DNA methylation. Methylation levels of maternally imprinted (*PEG1*, *PEG4*, *PEG5*, and *PEG10*), paternally imprinted (*H19*-IG DMR, *IGF2*-DMR0, and *MEG3*-IG DMR) regions, and obesity-related non-imprinted *HIF3A* gene were quantified by bisulphite pyrosequencing in sperm samples of 294 human donors undergoing *in vitro* fertilization or intracytoplasmic sperm injection, and in 113 FCBs of the resulting offspring. Multivariable regression analyses revealed that *MEG3* intergenic differentially methylated region (IG DMR) showed positive correlation between sperm methylation and donor’s BMI. A gender-specific correlation between paternal BMI and FCB methylation was observed for *MEG3*-IG DMR, *HIF3A*, and *IGF2*-DMR0. The former two genes displayed same directional nominal association (as sperm) between paternal BMI and FCB methylation in male offspring. Hypomethylation of *IGF2*-DMR0 with increased paternal BMI was observed in FCBs from female offsprings. Our results suggest that male obesity is nominally associated with modification of sperm DNA methylome in humans, which may affect the epigenome of the next-generation. Nevertheless, it is important to note that none of the associated p-values survived multiple testing adjustments. Future work should test the effect of associated methylation aberrations in the offspring as DNA methylation was shown to control expression and/or imprint establishment across the studied genes.

## Introduction

According to the World Health Organization (WHO), the worldwide prevalence of obesity has nearly doubled since 1980. Obesity is viewed as a complex multifaceted disease with a wide range of determinants including behavioral and heritable causes [1]. The estimated heritability of obesity is approximately 75% nevertheless the identified genetic variants so far can explain less than 30% of individual body mass index (BMI) levels and weight variation [2]. Recently, several research efforts focused on determining whether there is an epigenetic basis influencing the risk for metabolic disorders [3]. A large multicenter epigenome-wide association study (EWAS) has identified increased methylation at the hypoxia-inducible factor 3 alpha (*HIF3A*) gene in blood and adipose tissue to be associated with increased BMI [4]. Furthermore, a variably methylated region in the pro-opiomelanocortin gene (*POMC*) was reported to regulate human body weight [5].

The environmental influence experienced by an individual can be transmitted to the next-generation and have an effect on the offspring’s phenotype [6, 7]. It is recently becoming clear that parental obesity has a negative influence on the health and well-being of the offspring. Children born to obese mothers are well documented to have an increased predisposition for obesity, insulin resistance, and metabolic perturbations in later life [8]. These increased risks are not limited to maternal influences as epidemiological evidence has also shown that the offspring of obese fathers are at a higher risk of developing metabolic disorders [9, 10]. Newborn males of obese fathers exhibit a significant increase in birth weight as well as a change in several anatomical parameters including head circumference, abdominal diameter, and abdominal circumference [9]. Furthermore, a child born to an obese father and normal weight mother showed an increased risk of becoming obese whereas an obese mother and normal weight father could not predict whether the child would be overweight or obese [10]. Paternal effects were additionally reported to have transgenerational influences where an increase in nutritional abundance was significantly associated with increased rates of diabetes and cardiovascular problems in the children and grandchildren [11, 12]. Elegant rodent studies provide evidence for non-genetic germline transmission of obesity and insulin resistance in the progeny of mice fed a high fat diet [13]. Fathers exposed to nutritional perturbations including high fat diet, low protein diet, and caloric restriction were reported to sire offspring with pancreatic β-cell dysfunction and impaired glucose-insulin homeostasis [14–16]. In humans, recent reports have shown that paternal obesity and aging can epigenetically reprogram the gametes and that these epigenetic alterations might be subsequently transmitted to the children [17, 18]. For instance, weight loss after bariatric surgery was shown to remodel sperm DNA methylation patterns particularly in regions/genes that play a role in appetite control [19].

Imprinted genes are known to escape the wave of methylation reprogramming during early embryogenesis and have been proposed as carriers of epigenetic information into the next-generation [20]. As a result, any epigenetic alterations at imprinted genes can be transmitted via the germ cells thus contributing to future disease risk. Imprinted loci show a parent-of-origin dependent expression regulated by epigenetic silencing of either the maternal or paternal allele. The methylation marks are stably inherited via the germline ensuing mono-allelic expression in all somatic tissues [21]. Imprinted genes are well-known to control fetal growth where the parental ‘conflict theory’ proposes that paternally expressed genes (PEGs) promote nutrition acquisition from the mother whereas maternally expressed genes (MEGs) limit the usage of resources by the offspring [22]. Imprinted genes are also known to play an important role in a wide array of biological processes and diseases including intrauterine growth restriction (IUGR), obesity, and diabetes mellitus [23].

To study male obesity associated changes in sperm and their epigenetic transmission into the next-generation, we performed an in-depth methylation analysis at the regulatory regions of four maternally imprinted genes, three paternally imprinted loci, as well as the hypoxia inducible factor 3 alpha subunit (*HIF3A*) in a large cohort of 294 human sperm samples undergoing *in vitro* fertilization (IVF) or intracytoplasmic sperm injection (ICSI) as well as in the collected fetal cord blood (FCB) of their respective offspring when a live birth was achieved.

## Materials and methods

### Ethics approval and consent to participate

The Ethics committee at the medical faculty of the University of Würzburg approved this study on human sperm and fetal cord blood samples (no. 117/11 and 212/15). Written informed consent was collected from all participating couples.

### Study samples

The left-over swim-up sperm fractions (excess material) after IVF/ICSI were collected at the Fertility Center Wiesbaden, pseudonymized and frozen at -80°C until further use. In this study, the human sperm cohort consisted of a total of 294 samples including sperm from 103 males where a live birth was achieved after assisted reproductive technology (ART). We were successful in acquiring the fetal cord blood samples from collaborating obstetric clinics throughout Germany. Following live birth, a total of 113 FCB samples (including 10 pairs of twins) were collected from new-borns after a successful IVF/ICSI.

### Genomic DNA isolation and sodium bisulphite conversion

After thawing, the swim-up sperm fraction was purified by Silane-coated silica density gradients PureSperm 40/80 (Nidacon, Mölndal, Sweden). Purified sperm was resuspended in 300 μl buffer whose stock consisted of 5 ml of 5 M NaCl, 5 ml of 1 M Tris-HCl (pH 8), 5 ml of 10% SDS (pH 7.2), 1 ml of 0.5 M EDTA (pH 8), 1 ml of 100% β-mercaptoethanol and 33 ml of dH_2_O. Upon addition of 100 μl proteinase K (20mg/ml), the samples were initially incubated for 2 h at 56°C (on a thermomixer). Additional 20 μl proteinase K was added and samples were incubated for another 2 h at 56°C. Subsequently, sperm DNA was isolated using the DNeasy Blood and Tissue kit (Qiagen, Hilden, Germany) following the recommendations of the manufacturer. The FlexiGene kit (Qiagen, Hilden, Germany) was used for FCB DNA isolation. The concentration and quality of DNA were measured with the NanoDrop 2000c spectrophotometer (Thermo Scientific, Massachusetts, USA). Bisulphite conversion of sperm and FCB DNA (1 μg) was performed using the EpiTect Fast 96 Bisulphite kit (Qiagen, Hilden, Germany) following the manufacturer’s protocol and the converted DNA was stored at -20°C. In our experience, the average methylation variation between the technical replicates (including bisulphite conversion, PCR, and pyrosequencing) is approximately 1–2 percentage points.

### Bisulphite pyrosequencing

For primer design (S1 Table), the Pyro-Mark Assay Design 2.0 software (Qiagen, Hilden, Germany) was used. Methylation levels of four paternally expressed genes *PEG1*/mesoderm specific transcript (*MEST*), *PEG4*/small nuclear ribonucleoprotein polypeptide N (*SNRPN*), *PEG5*/neuronatin (*NNAT*), and *PEG10*/epsilon sarcoglycan (*SGCE*), three paternally imprinted loci *H19* intergenic differentially methylated region (IG DMR), insulin like growth factor 2 (*IGF2*) differentially methylated region 0 (DMR0), and *MEG3*-IG DMR, and one non-imprinted gene *HIF3A* were quantified. Assays were established by using methylation standards with 0%, 25%, 50%, 75%, and 100% methylation. Polymerase chain reaction (PCR) for each sample was performed in 25 μl reaction consisting of 2.5 μl 10x PCR buffer with MgCl_2,_ 0.5 μl (10mM) dNTPs, 1.25 μl (10pmol/ml) of each reverse and forward primer, 0.2 μl (5 U/μl) FastStart Taq DNA polymerase (Roche Diagnostics, Mannheim, Germany), 1 μl (~25 ng) bisulphite converted DNA and 18.3 μl dH_2_O. Pyro-sequencing was carried out using Pyro Q-CpG software (Qiagen, Hilden, Germany) and PyroMark Gold Q96 CDT reagent kit on the PyroMark Q96 MD system. Unmethylated and fully methylated DNA standards (Qiagen, Hilden, Germany) were used as controls in each pyro-sequencing run.

### Statistical analysis

Statistical analyses have been performed with the computational statistical software R (version 3.2.2). The methylation measurements (beta values) for the imprinted genes in haploid cells (sperm) are usually close to the border of the possible ranges in the standard unit interval (0; 1). To properly model this data, a multivariable regression analysis using beta model has been applied as implemented in the R package ‘betareg’ (version 3.1). As described in detail [24], this approach inherently allows to model heteroskedasticity or skewness which is often observed in data with values in the standard unit interval (e.g. rates or proportions) such as methylation rates. For the beta model, we used the default logit link and thus estimates can be back-transformed using the inverse logit function. Due to the logit link function, the estimates of the beta regression can be interpreted similar to logistic regression models and thus denote the additional increase (or decrease in case of a negative coefficient) in the log-odds of observing an allele in a methylated state. Average sperm DNA methylation values (the dependent variable in the model) of each amplicon have been regressed against donor’s BMI adjusting for potential confounding factors, which have been selected based on known factors influencing DNA methylation. The multivariable beta regression model for the sperm methylation data contained donor’s BMI and age, sperm concentration and whether or not a live birth was achieved after ART.

For FCB data with methylation values of imprinted genes in the mid-range, multivariable regression analysis using classical linear models were used. Multiple testing correction for false discovery rate (FDR) has been performed using the method of Benjamini and Hochberg. The regression models included birth weight and gestational week of the child, paternal and maternal age, paternal and maternal BMI, and gender of the child including the interaction between paternal as well as maternal BMI and child’s gender to allow for a varying effect of paternal and maternal BMI between the FCB genders.

## Results

### Characteristics of samples in the study

The body mass index (BMI) was calculated from the measured weight (in kilograms) and height (in centimeters) of the participating individuals. Based on the WHO guidelines of BMI categorization for adults, out of 294 sperm samples, one sample (0.34%) with 17.30 BMI fell in ‘underweight’ class; 144 samples (48.97%) in ‘normal weight’ class (BMI: 19.00–24.90); 149 samples (50.68%) in ‘Pre-obesity/obesity’ class (BMI: 25.00–40.30). To be more specific, 120 samples were ‘Pre-obese’ (BMI: 25.00–29.90) and the remaining 29 samples were classified as obese (BMI: 30.00–40.30). The FCB study cohort consisted of a total of 113 collected FCB samples (including 10 pairs of twins) from offspring fathered by 103 of the 294 sperm donors after a successful IVF/ICSI. Among them, 54 samples (47.79%) were female and 59 samples (52.21%) were male. Birth weight significantly correlated with gestational week of the child (rho = 0.67, p < 0.0001). Father’s age (103 out of 294 samples) significantly correlated with mother’s age (rho = 0.56, p < 0.0001). Clinical parameters of the sperm and fetal cord blood study samples are summarized in Table 1.

**Table 1:** Clinical parameters of the sperm and fetal cord blood samples.

Parameters	Sperm samples	Fetal cord blood samples
Sample size (N)	294	113
Sex of the child (Female; Male)	-	47.79% F (n = 54); 52.21% M (n = 59)
Paternal BMI (kg/m^2^) (range; mean ± SD)	17.30–40.30; 25.75 ± 3.1	17.30–40.30; 25.78 ± 3.3
Paternal age (years) (range; mean ± SD)	25.71–65.82; 38.95 ± 5.9	28.08–52.85; 38.63 ± 5.1
Sperm concentration (million/ml) (range; mean ± SD)	0.20–210.0; 31.81 ± 36.32	0.20–200.0; 35.99 ± 39.97
Maternal BMI (kg/m^2^) (range; mean ± SD)	-	17.00–39.80; 23.19 ± 4.0
Maternal age (years) (range; mean ± SD)	-	22.08–42.68; 34.62 ± 3.7
Birth weight (grams) (range; mean ± SD)	-	1510.00–4950.00; 3154.17 ± 602.1
Gestational weeks (range; mean ± SD)	-	34–42; 39.53 ± 1.8

The relevant demographics that were considered in the regression models for the analyzed sperm and fetal cord blood samples.

### Sperm DNA methylation signatures correlating with donor’s BMI

Imprinted genes are the best candidates to study inter-generational epigenetic inheritance since they bypass the genome wide epigenetic reprogramming mechanism occurring after fertilization. In this regard, we selected four maternally imprinted genes (*MEST*/*PEG1*, *SNRPN*/*PEG4*, *NNAT*/*PEG5*, and *SGCE*/*PEG10*), three paternally imprinted loci (*H19*-IG DMR, *IGF2* DMR0, and *MEG3*-IG DMR) and one obesity related gene *HIF3A* to check the correlation of male BMI with sperm DNA methylation. To determine the average methylation levels of these loci, bisulphite pyrosequencing was performed. The number of CpGs quantified for each gene is provided in S1 Table. For sperm, multivariable regression analysis using beta model was applied in order to adjust for potential confounding factors. Because of limited amounts of DNA, we analyzed 261 sperm samples for *MEST*, 263 for *SNRPN*, 262 for *NNAT*, 261 for *PEG10*, 255 for *H19-IG* DMR, 271 for *IGF2* DMR0, 265 for *MEG3-IG* DMR, and 260 for *HIF3A*. *MEG3*-IG DMR sperm DNA methylation showed a positive correlation (nominal significance without multiple testing adjustment) with male BMI (regression estimate +0.014, P = 0.043). Table 2 shows the result of the regression models when sperm DNA methylation was correlated with donor’s BMI. In additional S1 File, we provide statistics without adjusting to any confounding factors.

**Table 2:** Multivariable regression analysis using beta model: sperm DNA methylation in relation to donor’s body mass index.

Amplicon	Estimate	Standard error	p value
*MEST* (n = 261)	-0.004	0.009	0.661
*SNRPN* (n = 263)	-0.001	0.009	0.939
*NNAT* (n = 262)	-0.010	0.010	0.324
*PEG10* (n = 261)	-0.001	0.011	0.936
*H19*-IG DMR (n = 255)	0.012	0.009	0.191
*IGF2* DMR0 (n = 271)	0.003	0.005	0.632
***MEG3*-IG DMR** (n = 265)	**0.014**	**0.007**	**0.043**
*HIF3A* (n = 260)	0.008	0.004	0.080

The correlation of donor’s body mass index (kg/m^2^) with DNA methylation (%) of eight analyzed amplicons in sperm. The model was adjusted for sperm concentration, donor’s age, and ART success.

### Transmission of paternal BMI effects from sperm to next-generation in a gender specific manner

**Fig 1:**
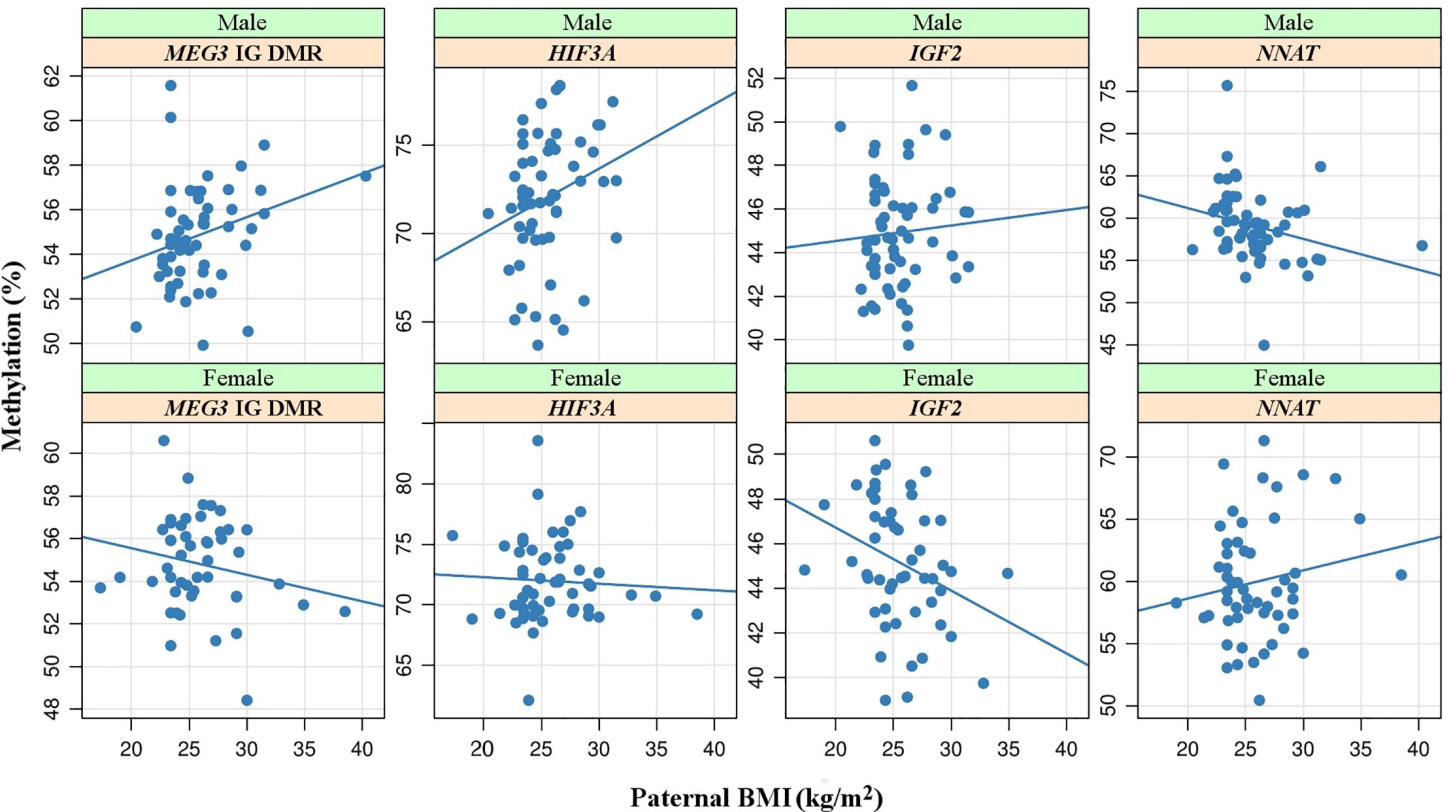
Correlation of paternal BMI with cord blood DNA methylation. Scatter plots showing the correlation between *MEG3*-IG DMR, *HIF3A*, *IGF2*, and *NNAT* methylation and the paternal BMI in male fetal cord blood (upper panel) and female fetal cord blood (lower panel) samples. Each dot in the plot represents the average methylation of several targeted CpGs in an individual cord blood sample after measurement by bisulphite pyrosequencing. Regression line suggests direction of correlation with paternal BMI. RE on each plot indicates the regression estimate.

To examine the probable transmission of BMI induced sperm epigenetic signatures to the next-generation, we performed bisulphite pyrosequencing on 113 fetal cord blood samples which were retrieved after successful live birth. Similar to sperm samples, bisulphite pyrosequencing was also performed in the above-mentioned genes on the cord blood DNA. For FCB, multivariable regression analysis using linear models was used to adjust for potential confounding factors. Because of limited amounts of DNA, we analyzed 111 cord blood samples for *MEST*, 109 for *SNRPN*, 112 for *NNAT*, 113 for *PEG10*, 110 for *H19-IG DMR*, 110 for *IGF2 DMR0*, 103 for *MEG3-IG* DMR, and 112 for *HIF3A*. Interestingly, the influence of father’s BMI on the FCB DNA methylation varied depending on the gender of the child. Here, *MEG3*-IG DMR (regression estimate +0.002, P = 0.020) and *HIF3A* (regression estimate +0.004, P = 0.041) cord blood DNA methylation of males but not females showed a positive correlation with the BMI of the father. In addition, *IGF2* DMR0 (regression estimate -0.003, P = 0.034) showed a negative correlation with paternal BMI in female FCBs (Table 3 and Fig 1). However, none of the correlated genes survived multiple testing adjustment. It is noteworthy to mention that the methylation levels of none of the eight studied genes correlated with maternal BMI (Table 4). In additional S1 File, we present statistics without adjusting to any confounding factors. We additionally tested the extent to which DNA methylation in sperm correlates with cord blood methylation (additional S2 File). Here, the *MEG3-IG* DMR revealed the strongest correlation (p-value = 0.070) between methylation values across both studied tissues.

**Table 3:** Multivariable regression analysis using linear models: Fetal cord blood DNA methylation in relation to paternal body mass index.

Amplicon	FCB_Gender	Estimate	Standard error	p value
*MEST*	Female (n = 53)	0.003	0.002	0.296
	Male (n = 58)	0.002	0.003	0.409
*SNRPN*	Female (n = 51)	-0.001	0.001	0.276
	Male (n = 58)	-0.001	0.001	0.375
*NNAT*	Female (n = 53)	0.003	0.002	0.111
	Male (n = 59)	-0.003	0.002	0.104
*PEG10*	Female (n = 54)	-0.001	0.001	0.305
	Male (n = 59)	0.001	0.001	0.308
*H19*-IG DMR	Female (n = 52)	-0.004	0.003	0.159
	Male (n = 58)	0.002	0.003	0.447
***IGF2* DMR0**	**Female** (n = 52)	**-0.003**	**0.001**	**0.034**
	Male (n = 58)	0.000	0.002	0.809
***MEG3*-IG DMR**	Female (n = 48)	0.000	0.001	0.568
	**Male** (n = 55)	**0.002**	**0.001**	**0.020**
***HIF3A***	Female (n = 54)	-0.001	0.001	0.554
	**Male** (n = 58)	**0.004**	**0.002**	**0.041**

**Table 4:** Multivariable regression analysis using linear models: Fetal cord blood DNA methylation in relation to maternal body mass index.

Amplicon	FCB_Gender	Estimate	Standard error	p value
*MEST*	Female (n = 53)	0.002	0.002	0.426
	Male (n = 58)	-0.001	0.002	0.696
*SNRPN*	Female (n = 51)	0.000	0.001	0.890
	Male (n = 58)	-0.001	0.001	0.504
*NNAT*	Female (n = 53)	0.000	0.001	0.943
	Male (n = 59)	-0.001	0.002	0.707
*PEG10*	Female (n = 54)	0.000	0.001	0.544
	Male (n = 59)	0.001	0.001	0.537
*H19*-IG DMR	Female (n = 52)	0.000	0.002	0.901
	Male (n = 54)	-0.002	0.002	0.337
*IGF2* DMR0	Female (n = 52)	-0.001	0.001	0.331
	Male (n = 58)	0.001	0.001	0.377
*MEG3*-IG DMR	Female (n = 48)	0.000	0.001	0.644
	Male (n = 55)	0.000	0.001	0.644
*HIF3A*	Female (n = 54)	0.000	0.001	0.825
	Male (n = 58)	0.000	0.001	0.978

The correlation of father’s body mass index (kg/m^2^) with DNA methylation (%) of eight analyzed amplicons in next-generation fetal cord blood samples. The model included birth weight and gestational week of the child, paternal and maternal age, maternal BMI, and gender of the child including the interaction between paternal BMI and child’s gender to allow for a varying effect of paternal BMI between the FCB genders.

The correlation of mother’s body mass index (kg/m^2^) with DNA methylation (%) of eight analyzed amplicons in next-generation fetal cord blood samples. The model included birth weight and gestational week of the child, paternal and maternal age, paternal BMI, and gender of the child including the interaction between maternal BMI and child’s gender to allow for a varying effect of maternal BMI between the FCB genders. None of the analyzed amplicons showed association between cord blood methylation and maternal BMI.

## Discussion

In this study, we measured whether an increased BMI can lead to methylation changes in male sperm, which might subsequently alter the DNA methylome of the next-generation. We focused on studying potential epigenetic inheritance at imprinted genes whose DNA methylation is stably maintained through the genome-wide reprogramming phase occurring after fertilization. We quantified methylation changes in a large cohort of >290 sperm samples and in 113 fetal cord bloods. It is generally the methylation density of several contiguous CpGs in a cis-regulatory region rather than methylation at individual CpG site that turns a gene on or off [25, 26]. In line with this, the designed pyrosequencing assays for all the genes systematically analyzed the methylation status of several CpGs across many thousand DNA molecules per samples. After adjusting for potential confounding factors, our analysis revealed the paternally imprinted region *MEG3*-IG DMR as one of the genes whose methylation increased (p-value = 0.043) in sperm due to an increase in the BMI levels of the donor. A similar effect was also observed in male offspring fathered by obese males where increased paternal BMI was associated with an increased methylation at *MEG3*-IG DMR (p-value = 0.020). The *MEG3*-IG DMR is positioned at the intergenic region between the maternally expressed gene 3 (*MEG3*) and delta-like 1 homolog (*DLK1*). The intergenic DMR serves as the imprinting control region for the *DLK1*-*MEG3* cluster and regulates gene expression and imprinting during development [27]. Recently, it has been shown that microRNAs in the *DLK1*-*MEG3* gene cluster are dramatically downregulated due to increased methylation at the *MEG3*-DMR in pancreatic islets of Type 2 diabetes mellitus patients [28]. The long non-coding RNA *MEG3* is also downregulated during adipogenesis and gene knockdown was shown to promote adipogenic differentiation [29].

Furthermore, we identified that DNA methylation levels of the obesity-related gene *HIF3A* showed a positive correlation with father’s BMI in the cord blood of male children (p-value = 0.041). Previously, a large epigenome-wide association study of DNA methylation in adult whole blood and adipose tissue (unlike sperm and cord blood in the present study) identified a significant correlation between BMI and methylation at three CpG sites in the first intron of *HIF3A* [4]. Several studies replicated the aforementioned association at the *HIF3A* locus across different tissues including umbilical cord DNA of newborns [30–33]. While one study reported that the association between the blood DNA methylation of *HIF3A* gene and donor’s BMI was stronger in young-aged population subset (≤ 66 years) [34], another report did not find any correlation between the two [35]. The result of our present study contradicts with the associations observed between parental BMI and offspring *HIF3A* methylation in a study by Richmond *et al*, 2016. [36]. Here, the authors measured methylation using the Infinium HM450 BeadChip array on ~1000 samples and examined associations at the individual CpG level, however they did not implement any gender-specific testing in the offspring [36]. Furthermore, we measured methylation in cord blood DNA of children born after IVF/ICSI, which can be an additional confounding factor in our study. It is important to emphasize that previous reports, in addition to the current study, only tested for associations between *HIF3A* methylation and BMI levels, which do not indicate the causality of *HIF3A* methylation alterations to obesity. Similar to the previous studies in human blood [34–36] and in sperm [19], the data presented support the idea that DNA methylation (of *HIF3A* and other genes) was an effect rather than a cause of increased BMI.

We also observed a negative correlation (p-value = 0.034) between *IGF2* methylation in female cord blood and paternal BMI. A similar association between *IGF2* hypomethylation and paternal obesity was previously reported in neonates enrolled in the Newborn Epigenetics Study cohort [37]. As shown in Fig 1, DNA methylation at the *MEG3* IGDMR, *HIF3A*, *IGF2*, and *NNAT* exhibited a correlation with paternal BMI however in opposite directions when comparing male and female cord bloods. The gender-specific effects observed are in line with several previously published studies [16]. In rodents, Huypens *et al*. observed gender-specific effects on body-weight gains and insulin levels in mice fathered by males fed a high-fat diet [13]. Similar gender-related differences were reported in other studies investigating the influences of parental obesity or nutrition on the offspring [9, 16, 38]. Here, we would like to emphasize that none of the reported p-values in the present study (both in sperm and in cord blood) survived adjustment for multiple testing, exhibiting only nominal significance to male BMI. This indicates that the correlations displayed are weak which might be due to the low power of the study. Consistent with earlier studies [37, 39], the observed paternal BMI effects in FCB were of small effect size. Most likely it is the sum of multiple small methylation changes, which transmits the disease susceptibility associated with adverse parental factors to the next-generation.

Cord blood DNA methylation levels in none of our studied genes exhibited a significant correlation with maternal BMI values, which contrasts with another study [40]. Here, the authors systematically meta-analyzed the correlations between pre-conceptional maternal BMI and DNA methylation sites (450K) in the cord blood of the offspring. After adjusting for cell proportions, the number of genome-wide significant CpGs was reported to be only 104, however with a very small effect size/methylation variation [40]. Most importantly, none of the analyzed imprinted regions from our study harbored a differentially methylated CpG with genome-wide significance, thus possibly indicating that maternal BMI is not influencing methylation at the studied imprinted genes in cord blood DNA. As spermatogonial stem cells divide constantly to self-renew and differentiate, one might speculate that sperm cells are more prone to epigenetic errors during cell division in contrast to oocytes, which are arrested at an early stage of the first meiotic division. It is noteworthy to mention that the cord blood DNA methylation measurements performed in this study and in all previous studies represent the average methylation of both parental alleles. Paternal BMI effects on the paternal allele may be covered up by variation of maternal allele methylation or vice versa. Ideally, one would like to separately delineate paternal versus maternal allele methylation in somatic tissues of the offspring.

Male infertility and low-quality spermiogram parameters are the clinical factors frequently associated with methylation alterations at imprinted genes in sperm [41, 42], which is one limitation associated with our study cohort. Nevertheless, we have corrected for ART success and analyzed methylation in a large number of samples, which allowed us to delineate minor methylation changes linked to obesity in sperm. A recent report by Soubry *et al*. identified methylation abnormalities at several imprinted genes in sperm of 23 overweight/obese fertile males when compared to 44 fertile controls [17]. In their study, *MEG3*-IG DMR was similarly reported to have an increased methylation in the sperm of obese males, which is in line with our findings. Furthermore, this confirms that the identified DNA methylation changes at *MEG3*-IG DMR are linked to obesity since they also occur in fertile males. Our study additionally provides evidence for a gender-specific positive correlation in *MEG3*-IG DMR between male cord blood methylation and father’s BMI indicating possible inheritance of methylation signatures. Several studies have reported an association between methylation changes at imprinted genes in the fetus and exposure to maternal smoking during development [43]. It is important to note that we did not include parental smoking as a confounding factor in our multivariate regression analysis as we do not have reliable information regarding smoking habits in our studied cohort.

## Conclusions

In this study, we focused on epigenetic changes in sperm in relation to donor’s BMI and whether these changes can be transmitted to the offspring. We identified subtle methylation alterations correlating with donor’s BMI in male sperm at the paternally imprinted *MEG3*-IG DMR locus. A gender-specific correlation between the DNA methylation of *MEG3*-IG DMR, *HIF3A*, and *IGF2* DMR0 in the cord blood of the offspring and paternal BMI was observed. Our results indicate that obesity in males can influence DNA methylation programming in sperm and also affect the epigenome of the next-generation. These findings are in line with earlier research in humans [17, 19, 37, 39]. Epigenetic inheritance might help explain the escalating incidence of obesity worldwide, which cannot be attributed to genetic factors on their own. Nevertheless, it remains to be shown whether those changes can influence the risk of developing obesity in the offspring during adulthood. A longitudinal follow-up of our studied cohort is needed to help answer this question.

## Supporting information

S1 Table PCR and sequencing primers used for bisulphite pyrosequencing. S1 Table shows the forward and reverse primers for polymerase chain reaction and bisulphite pyrosequencing for all the eight amplicons analyzed in this study. Primers indicated by a star are biotinylated at the 5’ end and the chromosomal location is based on Ensembl release 89.
(DOC)

S1 File DNA methylation correlations without adjusting to confounding factors. S1 File shows the methylation statistics when DNA methylation is correlated with BMI without adjusting to any confounding factors.
(XLS)

S2 File DNA methylation correlation between sperm and cord blood. S2 File shows the spearman's rank correlation of DNA methylation levels in sperm and corresponding cord blood samples.
(XLS)

## Abbreviations

ART: Assisted Reproductive Technology
BMI: body mass index
DLK1: delta-like 1 homolog
dNTPs: deoxyribose nucleoside triphosphates
EDTA: ethylenediamine tetra acetic acid
EWAS: epigenome wide association study
FCB: fetal cord blood
FDR: false discovery rate
HCl: hydrogen chloride
HIF3A: hypoxia inducible factor 3 alpha
HNA: hypermethylation of the non-imprinted allele
ICSI: intracytoplasmic sperm injection
IG DMR: intergenic differentially methylated region
IGF2: insulin like growth factor 2
IUGR: intrauterine growth restriction
IVF: *in vitro* fertilization
MEGs: maternally expressed genes
MEST: mesoderm specific transcript
MgCl_2_: magnesium chloride
NaCl: sodium chloride
NNAT: neuronatin
PCR: polymerase chain reaction
PEGs: paternally expressed genes
POMC: pro-opiomelanocortin gene
SDS: sodium dodecyl sulfate
SGCE: epsilon sarcoglycan
SNRPN: small nuclear ribonucleoprotein polypeptide N
WHO: world health organization
