# Supplementary material for: Male obesity effects on sperm and next-generation cord blood DNA methylation
Source: PLoS One. 2019 Jun 27;14(6):e0218615. doi: 10.1371/journal.pone.0218615 (PMC6597061; doi:10.1371/journal.pone.0218615)
Supplement: S1 Table — shows the forward and reverse primers for polymerase chain reaction and bisulphite pyrosequencing for all the eight amplicons analyzed in this study. Primers indicated by a star are biotinylated at the 5’ end and the chromosomal location is based on Ensembl release 89. (DOC) [file pone.0218615.s001.doc]

| **Gene** | **Primer** | **Sequence (5'-3')**^a^ | **Location**^b^ | **No. of CpGs** |
| --- | --- | --- | --- | --- |
| *MEST* | Forward | GATTTAAAGGATAGGTTTTAGTAT | Chr 7: 130,492,924 – 130,493,220 |  |
|  | Reverse | *AACCAAAATAAACAATCCCTAC |  |  |
|  | Sequencing | GTATTTTTTAGATTTTAGTAATAAG |  | 4 |
| *SNRPN* | Forward | *AGGGAGTTGGGATTTTTGTATT | Chr 15: 24,954,865 – 24,955,102 |  |
|  | Reverse | CCCAAACTATCTCTTAAAAAAAAC |  |  |
|  | Sequencing 1 | ACACAACTAACCTTACCC |  | 3 |
|  | Sequencing 2 | CCAACCTACCTCTAC |  | 3 |
| *NNAT* | Forward | GGATTTTGTTTTAAAATGGAGGGGTAT | Chr 20: 37,520,875 – 37,521,038 |  |
|  | Reverse | *CCATCCATCCCCAAAATAAACTT |  |  |
|  | Sequencing | TTTAAAATGGAGGGGTATT |  | 2 |
| *PEG10* | Forward | *GTGTTAAGGAGTTGGGAGGA | Chr 7: 94,657,812 – 94,657,994 |  |
|  | Reverse | TCTACAACCCTATAACAACCAATCTCA |  |  |
|  | Sequencing | CCTAATATACCTTCTCTA |  | 3 |
| *H19* | Forward | *TGGGTATTTTTGGAGGTTTTTTT | Chr 11: 1,999,845 – 2,000,061 |  |
|  | Reverse | ATAAATATCCTATTCCCAAATAA |  |  |
|  | Sequencing | ATATATAAACCTACACTACC |  | 3 |
| *IGF2* DMR0 | Forward | GGAGGGGGTTTTAGTAAAAGTTAT | Chr 11: 2,148,223 – 2,148,456 |  |
|  | Reverse | *TCCCAACCTCCCTAACACAAA |  |  |
|  | Sequencing | AGTAAAAGTTATTGGATATATAGT |  | 3 |
| *MEG3*-IG DMR | Forward | AGGGTTAGGAAGTTTAGTAGGTTA | Chr 14: 100,809,308 – 100,809,458 |  |
|  | Reverse | *ACTACTCCTTAAACAAAAAAACACATAAT |  |  |
|  | Sequencing | GTAGTAAATTAAAGTGTATTAGAGA |  | 5 |
| *HIF3A* | Forward | TGGTTGAAGGGTTATTTAGGG | Chr 19: 46,298,269 – 46,298,496 |  |
|  | Reverse | *ACTCTATCCCACCCCTTTT |  |  |
|  | Sequencing 1 | TTTAGGGGGTGTAGG |  | 7 |
|  | Sequencing 2 | GGTGAGATGATTTTATAGGAA |  | 1 |
|  | Sequencing 3 | GTTAAGAGGGGTTTTTATT |  | 3 |

**S1 Table. PCR and sequencing primers used for bisulphite pyrosequencing.**

^a^ Primers indicated by a star are biotinylated at the 5' end. ^b^ Chromosomal location based on Ensembl release 89 – May 2017
